# Succession of Ambrosia Beetles Colonizing the Logs of Fallen Alder and Birch Trees

**DOI:** 10.3390/insects13030223

**Published:** 2022-02-23

**Authors:** Yong Peng, Anut Buranapanichpan, Naoto Kamata

**Affiliations:** 1 Department of Forest Science, Graduate School of Agricultural and Life Sciences, The University of Tokyo, Bunkyo, Tokyo 113-8657, Japan; anut_b@uf.a.u-tokyo.ac.jp; 2 The University of Tokyo Hokkaido Forest, The University of Tokyo Forests, Graduate School of Agricultural and Life Sciences, The University of Tokyo, Furano, Hokkaido 079-1563, Japan; kamatan@uf.a.u-tokyo.ac.jp

**Keywords:** *Treptoplatypus severini*, *Heteroborips seriatus*, *Xylosandrus germanus*, *Xyleborinus attenuatus*, early- vs. late-successional species, niche, Scolytinae, Platypodinae

## Abstract

**Simple Summary:**

To determine the succession of ambrosia beetles after tree felling, two Betulaceae tree species, an alder (*Alnus hirsuta*), and a white birch (*Betula platyphylla* var. *japonica*) were felled as bait logs in central Hokkaido, Japan, in 2016. From 2016 to 2018, the bait logs were dissected late in each flying season to collect ambrosia beetles. During the period of monitoring, most beetles were collected during the first 2 years. The ambrosia beetle faunas colonizing the two plant species were found to be similar, and it was established that each beetle species appeared in the same sequence in each of the two plant species, albeit with differing temporal patterns. These observations thus indicate a similar sequence of beetle succession in the two tree species, and that the relative niches of beetle species in the successional sequence after tree felling can be determined using this methodology.

**Abstract:**

Ambrosia beetles bore into the xylem of woody plants, reduce timber quality, and can sometimes cause devastating damage to forest ecosystems. The colonization by different beetle species is dependent on host status, from healthy trees to the early stages of wood decay, although the precise factors influencing their host selection are not well known. Classic studies on plant ecology have determined the niches of different plant species in vegetation succession, based on comparisons of successions in different locations using ordination analyses, although the factors influencing the colonization of each species are largely undetermined. In this study, to characterize the succession of ambrosia beetles after tree felling, two Betulaceae tree species, an alder (*Alnus hirsuta*), and a white birch (*Betula platyphylla* var. *japonica*) were felled as bait logs in central Hokkaido, Japan, in 2016. From 2016 to 2018, the bait logs were dissected late in each flying season, and ambrosia beetles were collected from the logs. During the period of monitoring, the beetle colonization in both tree species was mostly concentrated in the first 2 years. We observed similarities in the beetle faunas colonizing the two plant species, and that individual species appeared in the same sequence in the logs of the two plant species, although the temporal patterns of colonization differed. Consequently, significant differences in beetle community compositions in the two host species were detected in each of the first 2 years of the study, whereas the difference in the overall composition of beetle assemblages (=pooled over 3 years) between the two plant species was smaller than that in either 2016 or 2017. We speculated that the differences in the temporal pattern of colonization could be attributable to differences in the rates at which the wood of the two tree species deteriorated. *Treptoplatypus severini* and *Xylosandrus crassiusculus* were considered to be early-successional species that commenced log colonization soon after felling, although *T. severini* has a wide niche and was collected during all 3 years of the study. Conversely, *Xyleborinus attenuatus* and *Heteroborips seriatus* were identified as probable late-successional species that showed a preference for older logs.

## 1. Introduction

Ambrosia beetles comprise a group of insects in the subfamilies Scolytinae and Platypodinae (Coleoptera: Curculionidae). These beetles carry the spores of ambrosia fungi that are subsequently released in galleries bored into the xylem of host trees, wherein they cultivate these symbiotic fungi on which the adults and larvae feed exclusively [1]. A number of ambrosia beetles can be vectors for pathogenic fungi that cause tree diseases. For example, the oak ambrosia beetle (*Platypus quercivorus*) carries the fungus *Raffaelea quercivora*, which causes Japanese oak wilt [2], and the redbay ambrosia beetle (*Xyleborus glabratus*) carries the fungus *R. lauricola*, which causes laurel wilt and results in the extensive mortality of trees belonging to the family Lauraceae in the southeastern United States [3]. In addition, ambrosia beetles, and their associated fungi, usually cause stains and degrade the valuable woody products [4].

Ambrosia beetles usually require host materials whose conditions are suitable for their symbiotic fungi. Most ambrosia beetle species colonize only the tissues of dying or recently deceased woody plants. In contrast, a very small number of ambrosia beetle species colonize healthy living trees [1,5]. Similarly, a small number of ambrosia beetle species exploit older plant material at a more advanced stage of decay [6]. Ambrosia beetles complete larval development within a selected host, and offspring beetles emerge from the brood tree in search of fresher host trees because the brood tree has become unsuitable for the development of the next generation. Therefore, locating and selecting a suitable host is critical to the reproductive success of ambrosia beetles [1,5]. In this regard, it has been established that these beetles generally discriminate suitable host trees from less suitable, extensively decayed, or overly colonized hosts, as well as non-hosts, via a range of olfactory responses to host volatiles and/or wood degradation products [7]. However, it is difficult to directly determine the factors that enable ambrosia beetles to recognize suitable hosts.

Ecological succession, the more or less predictable and orderly changes in the species composition or structure of an ecological community over time, has long been an intensively studied ecological phenomenon in vegetation science [8,9,10,11]. Given that vegetation is the dominant feature of landscapes, successional ecologists have, in the past century, focused primarily on vegetation succession and developed related concepts and methods [12], including ordination techniques, which have been widely used to analyze species distributions along environmental gradients and describe temporal changes in species composition [11,12,13,14]. Studies on vegetation succession have found that each plant species generally appears in a similar sequence during successional processes at different locations [9,10,11].

Recently, some studies have examined animal (mainly invertebrate) succession based on concepts and methods derived from the study of vegetation succession [15,16,17]. In this study, we investigated the succession of ambrosia beetles colonizing the logs of two tree species that were phylogenetically close to each other by using a methodology similar to that adopted in the classic studies of vegetation succession. According to existing research, closely related host trees are very likely to share similar herbivorous assemblages [18,19,20]. Therefore, we predicted that there would be similarities in the ambrosia beetle fauna colonizing the two tree species. Consistent with the classic studies on vegetation succession [9,10,11], we assumed that during the process of decay, the progression of ambrosia beetle invasion would occur in a similar sequence among different plant species. On the basis of this assumption, we also examined the relative niches of the different ambrosia beetle species (early- to late-successional species) in logs of the two tree species after felling.

## 2. Materials and Methods

### 2.1. Study Site Description

This experiment was conducted in the University of Tokyo Hokkaido Forest (UTHF), Furano City, central Hokkaido (43°10′–21′ N, 142°23′–41′ E, 190–1459 m a.s.l.) [21]. From 2016 to 2018, the mean annual temperature was 6.5 °C, with maximum and minimum temperatures of 35.1 °C and −24.3 °C, respectively (observatory point: 43°13′ N, 142°23′ E, 230 m a.s.l.). The mean annual precipitation was 1384 mm. The snow cover period usually ranges from the end of November until the beginning of April, with an average maximum snow depth of 83 cm [22,23,24]. This forest is a mixed forest within a transitional area from the cool temperate to sub-boreal zones. The dominant tree species are *Fraxinus mandshurica*, *Ulmus davidiana* var. *japonica*, *Alnus hirsuta*, and *Salix* spp. on the lower elevations (<300 m), *Abies sachalinensis* on the middle elevations (300–600 m), *Picea jezoensis*, *p. glehnii*, and *Betula ermanii* on the upper elevations (800–1200 m), and alpine vegetation (e.g., *Pinus pumila*) at the upper forest limit (>1200 m) [21].

### 2.2. Bait Log Preparation and Beetle Sampling

**Figure 1:**
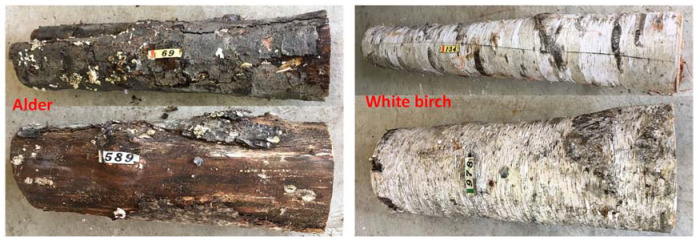
Logs of alder (**left**) and white birch (**right**) sampled in September 2018 (two years after being felled and exposed to beetle attack).

In this study, two Betulaceae tree species, an alder (AL, *Alnus hirsuta*), and a white birch (WB, *Betula platyphylla* var. *japonica*) were used as bait logs. In 2016, 24 healthy individuals of AL and WB were selected and felled in an AL (43°13′ N, 142°26′ E, 300 m a.s.l.) and WB plantation (43°13′ N, 142°26′ E, 300 m a.s.l.), respectively. The distance between the two plantations was approximately 1.5 km. To avoid missing the species that attack very fresh logs, felling times varied from May to July, because capturing flying beetles varies greatly depending on the seasonal occurrence of the beetle species (Peng Y et al., unpublished data, see also [25,26,27,28,29]). After removing all branches, the AL and WB logs were left on the ground in each plantation and exposed to insect attacks. Each log was divided into billets of almost the same length (approximately 100 cm) and tagged with numbered tape (Figure 1). Given that very few beetles were captured using ethanol-baited traps after late August (Peng Y et al., unpublished data), the first billet collection in each year occurred thereafter. In 2016, 129 billets of each tree species were cut three times between August and October. In 2017, billet collections occurred two times in August and October, with a total of 75 and 77 billets cut for AL and WB, respectively. In 2018, billets were sampled once in September, with 17 and 18 billets collected for AL and WB, respectively. The diameters of AL and WB billets ranged from 9.7 to 28.9 cm (18.3 ± 4.2, mean ± SD) and 9.8 to 32.5 cm (19.5 ± 5.2), respectively.

After each collection, billets were brought back to the UTHF nursery, where beetles in the logs were sampled by dissection. First, each billet was cut into smaller blocks of approximately 20 cm in length. Billets with a large diameter were split into smaller parts before cutting. Then, beetles under the bark were collected by gently removing the bark, and those in the sapwood were collected by cutting the block into smaller pieces. Each entry hole (gallery) was treated as a sampling unit. Beetles, including adults and/or larvae, collected from each active gallery/hole (a gallery/hole inhabited by beetles) were preserved in a 1.5-mL microtube containing 99.5% ethanol and maintained for further identification. Beetles belonging to the subfamilies Scolytinae and Platypodinae were identified. We initially sought the assistance of Dr. Roger A. Beaver in identifying selected individuals of each morphospecies, which served as reference material that was referred to when identifying the collected specimens.

### 2.3. Data Analysis

In this study, the beetles collected from each of the two tree species in each year were treated as a sample, and the analyses were based on the number of galleries, rather than individual beetles. Generalized linear models (GLMs) and zero-inflated count models, both with Poisson distribution, were fitted to examine the effects of tree species (SP) and sampling year (YR) on the number of active holes for all and each insect species. The best fitting model was selected using the Vuong test in the “pscl” package (ver. 1.5.5) [30] based on Bayesian information criterion (BIC) [31]. The results indicated that the zero-inflated Poisson (ZIP) model was the best fitting model. Multiple comparisons between YR were conducted using the Tukey method in the “multcomp” package (ver. 3.5.3) [32].

To determine the timing of the colonization of each insect species, we calculated the niche center ($N_{c}$) based on the species’ relative resource preference (the proportion at each resource state) using the following Equation:

(1) $$N_{c}=\sum^{\;}f_{i}x_{i}$$

where $f_{i}$ is the relative resource preferences of the species in the *i*th sampling year and $x_{i}$ is the position value of the *i*th sampling year [33]. The niche breadth ($B_{d}$) was measured as the uniformity of the species distributions among the resource states (sampling years or age of logs) using the Shannon–Wiener formula [34] in the package “spa” (ver. 0.2.2) [35]:

(2) $$B_{d}=-\sum^{\;}f_{i}lnf_{i}$$

When all individuals of a target species were associated with only a single-resource state (sampling year or age of logs), the niche breadth was zero. Generalized linear mixed models (GLMMs) were used to test the differences in the niche center and niche breadth of beetle assemblages between AL and WB, with tree species and insect species as the fixed and random effects, respectively. The effects of insect species on the niche center and niche breadth of beetles were also tested using GLMMs, with beetle species and tree species as the fixed and random effects, respectively. The GLMMs analysis was conducted using the “nlme” package (ver. 3.1-152) [36].

Indicator species analysis (INSPAN) was applied to identify the preferences of insect species for tree species and log age. The log age preference for AL and WB were identified separately. The correlation index was used to assess the association of the insect species with the tree species or log age, for which the statistical significance was evaluated via permutation tests [37]. The “indicspecies” package (ver. 1.7.9) was used for this analysis [37].

The difference in the beetle community compositions between tree species and different sampling years (or log age) was tested via a permutational multivariate analysis of variance (PERMANOVA) [38] using the “vegan” package (ver. 2.5-7) [39]. For the PERMANOVA, the Chao dissimilarity index was used to calculate the pairwise distances. Logs with no beetles were excluded from the analysis as they had no relevance when calculating pairwise dissimilarity. Nonmetric multidimensional scaling (NMDS), partnered with the Chao dissimilarity index, was employed to evaluate the relationship between the insect species, SP, and YR.

## 3. Results

**Figure 2:**
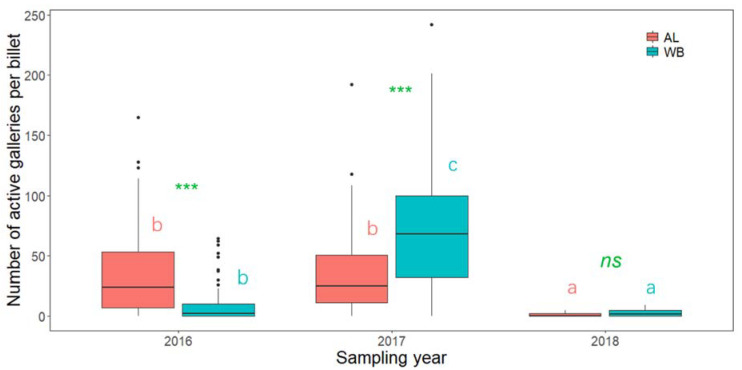
The number of active holes (galleries with beetles) per billet in logs of alder (AL) and white birch (WB) in different sampling years. The effects of the tree species and sampling year were tested using ZIP models. Different lowercase letters indicate a significant difference between the different sampling years for each tree species. *** indicate a significant difference between AL and WB in the same year at the 0.001 levels. *ns* indicates no significant differences between the tree species in the same sampling year.

The number of active holes (galleries with beetles) per billet was significantly influenced by SP and YR (Figure 2). The number of active holes in AL did not differ between 2016 and 2017, but both were greater than that in 2018. However, the number of active galleries in WB was significantly larger in 2017 than in either 2016 or 2018. The number of active holes was greater in AL than in WB in 2016, greater in WB than in AL in 2017, and did not differ between the two tree species in 2018. Accordingly, the beetles started to attack AL on a vast scale in 2016, with colonization continuing at a high rate in 2017, and there was a marked reduction in colonization in 2018. In comparison, a smaller number of beetles colonized WB logs in 2016, although a notable increase in colonization was detected in 2017, prior to a subsequent reduction in 2018.

Six ambrosia beetle species were collected from AL logs (Table 1). In addition to the six species found in AL, three other species of ambrosia beetle were also collected only from WB logs. The results showed that the beetle colonization of both tree species was mostly concentrated in 2016 and 2017. In 2018, only *Treptoplatypus severini* (Blandford) and *Xyleborinus attenuatus* (Blandford) were collected from the logs (predominantly the former). In 2016, the ambrosia beetle communities of both tree species were dominated by *T. severini* (78.0% and 96.3% in AL and WB logs, respectively). Besides *T. severini*, only a few individuals of other species were collected in WB logs in 2016, whereas the proportion of *Xylosandrus germanus* (Blandford) in AL logs reached 16%. In 2017, *Heteroborips seriatus* (Blandford) (52.1%) became the dominant species in WB, whereas the beetle assemblages in the AL logs continued to be dominated by *T. severini* (52.4%). Overall, in both AL and WB logs, the ambrosia beetle communities were mainly composed of *T. severini*, *H. seriatus*, *Xylosandrus germanus*, and *Xyleborinus attenuatus* (proportion >98%). The overall beetle assemblages in AL and WB logs were dominated by *T. severini* (68.7%) and *H. seriatus* (44.2%), respectively.

**Figure 3:**
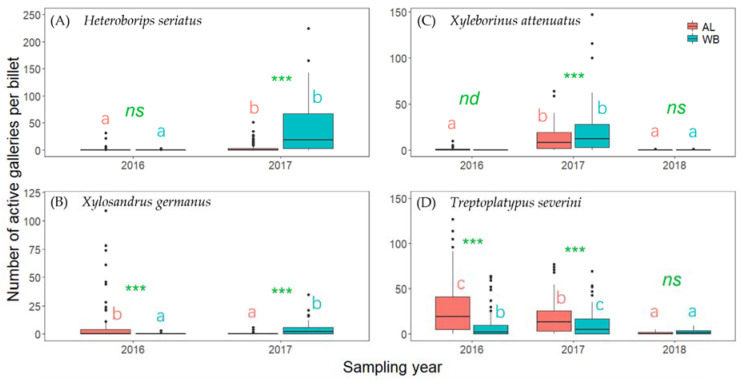
The number of active holes per billet of the four most abundant species found in alder (AL) and white birch (WB) logs in different sampling years ((**A**) *Heteroborips seriatus*, (**B**) *Xylosandrus germanus*, (**C**) *Xyleborinus attenuatus*, and (**D**) *Treptoplatypus severini*). Data for *H. seriatus* and *X. germanus* in 2018 are not shown, as no individuals were found in that year. The effects of the tree species and sampling year on the number of active galleries were tested using ZIP models. Different lowercase letters indicate that the number of active galleries for the same tree species differed significantly between the sampling years. *** indicate a significant difference in the number of active galleries between AL and WB in the same sampling year at the 0.001 level. *ns* indicates no significant differences in the number of active galleries between tree species in the same sampling year. *nd* indicates significance could not be determined as no *X. attenuatus* was sampled from WB logs in 2016.

The number of active holes per billet of the four most abundant species (*T. severini*, *H. seriatus*, *Xyleborinus attenuatus*, and *Xylosandrus germanus*) are shown in Figure 3. With regards to comparisons between the two host tree species, we detected no significant difference in the number of active galleries of *H. seriatus* between AL and WB in 2016, whereas in 2017 we recorded a greater number of occupied galleries in WB than in AL in 2017 (Figure 3A). Conversely, in the case of *X. germanus*, we detected a larger number of active galleries in AL than in WB in 2016 (Figure 3B), whereas the opposite trend was identified in 2017. Furthermore, with respect to *X. attenuatus*, no individuals were collected from WB in 2016, whereas we recorded greater numbers of active galleries in WB than AL in 2017, and no significant difference between tree species in 2018 (Figure 3C). The numbers of *T. severini* active holes recorded in 2016 and 2017 were significantly greater in AL than in WB, but did not differ in 2018 (Figure 3D). Regarding the comparison among sampling years, the number of *H. seriatus* active holes in both tree species was significantly greater in 2017 than in 2016 (Figure 3A). In AL, the number of *X. germanus* active holes was greater in 2016 than in 2017, whereas the opposite was true for WB (Figure 3B). In both tree species, the number of *X. attenuatus* active holes was significantly greater in 2017 than in either 2016 or 2018 (Figure 3C). In AL logs, the number of *T. severini* active holes showed a significant annual reduction from 2016 to 2018 (Figure 3D); in contrast, in WB logs, the number of *T. severini* active holes recorded in 2016 was smaller than that in 2017, although this was greater than that recorded in 2018.

**Figure 4:**
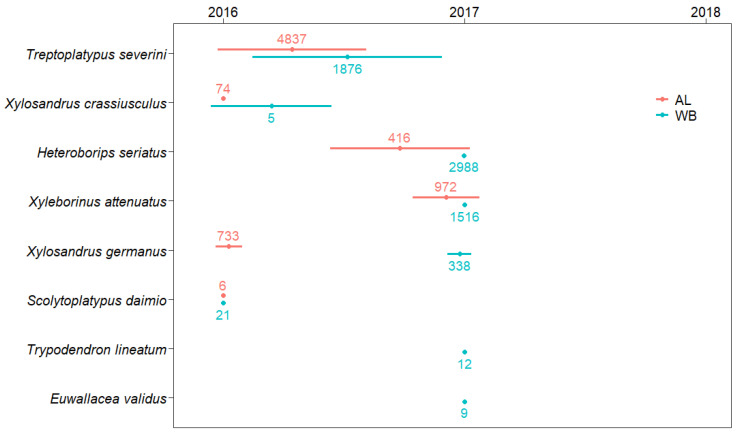
Niche center (dots) and breadth (bars) of the colonization timing of each insect species in the logs of alder (AL) and white birch (WB). The numbers indicate the total abundance (number of galleries with beetles) of each insect species. Here, the niche center ($N_{c}$) was calculated as the weighted mean of relative resource preference using the Equation: $N_{c}=\sum^{\;}f_{i}x_{i}$ [33], where $f_{i}\;$ is the relative resource preferences of the species in the *i*th sampling year and $x_{i}$ is the position value of the *i*th sampling year. The niche breadth ($B_{d}$ ) was measured as the uniformity of the species distribution among sampling years using the Shannon–Wiener formula: $B_{d}=-\sum^{\;}f_{i}lnf_{i}$ [34]. Niche breadth is minimized to zero when all individuals of a species are found in only one sampling year. *Scolytoplatypus mikado* was excluded because of the singleton. The results of the generalized linear mixed model (GLMM) indicated that the tree species and insect species both had marginally significant effects on the niche center (*p* = 0.063 and *p* = 0.061, respectively), but did not affect the niche breadth.

The niche center and breadth of the timing of individual beetle species colonization for both tree species are shown in Figure 4. The results of GLMMs indicated that tree species and beetle species had marginally significant effects on the niche center, although they had no appreciable influence with respect to niche breadth (Figure 4; Table 2). In the terms of host tree colonization, we established that the timing of beetle colonization of AL was slightly earlier than that of WB (*p* = 0.063). With respect to the comparison between beetle species, the colonization timing of *Xyleborinus attenuatus* was significantly later than that of *Xylosandrus crassiusculus*, and *S. daimio* (*p* < 0.05). Moreover, the niche center of each beetle species tended to differ between AL and WB, although it appeared to follow the same sequence.

**Figure 5:**
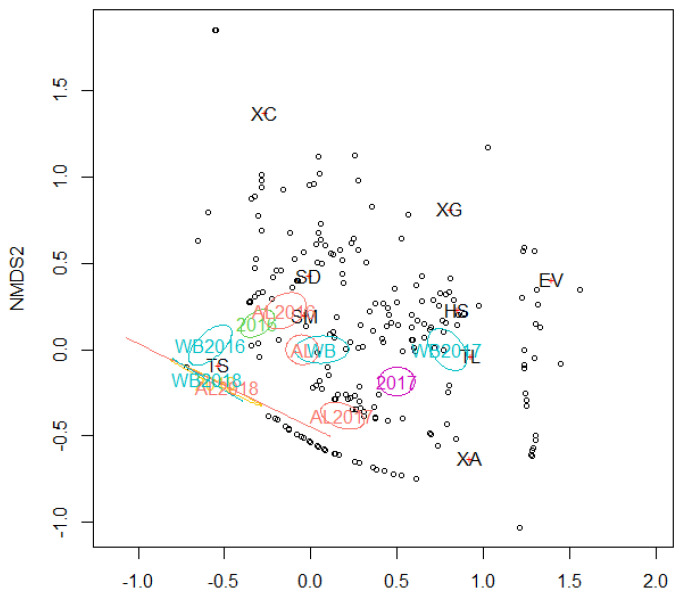
NMDS ordination of ambrosia beetle communities found in alder (AL) and white birch (WB) logs over three years. The ovals indicate a 95% confidence range of the regimes in each tree species, each year, or each of their combinations. Open circles (ο) and red crosses (+) indicate the billet samples and beetle species, respectively. The NMDS is based on Chao’s distance matrix. Logs without beetles were excluded from this analysis, as they are not relevant to Chao’s distance. See Table 1 for abbreviations of insect species.

The PERMANOVA results suggested that both SP and YR had significant effects on the community structure of the ambrosia beetle assemblages inhabiting AL and WB logs, with a significant interactive effect (SP: *R*^2^ = 0.0248, *p* < 0.001; YR: *R*^2^ = 0.3417, *p* < 0.001; and SP * YR: *R*^2^ = 0.1126, *p* < 0.001; Table 3). The NMDS results indicated that the difference in the overall community structure between AL and WB was small, but increased throughout each sampling year, except for 2018 (Figure 5). In each tree species, the beetle assemblages for each year were separate from each other (Figure 5), indicating that YR significantly affected the community structures in AL and WB.

The INSPAN results revealed that *X. crassiusculus* and *X. germanus* exhibited a preference for 1-year-old AL logs (Table 4). No insect species preferred 1-year-old WB logs. *Heteroborips seriatus* and *X. attenuatus* showed a preference for two-year-old AL and WB logs. *Xylosandrus germanus* and *T. lineatum* preferred two-year-old WB logs. *Treptoplatypus severini* was the indicator species in both tree species for the first two years after felling. No beetle species showed a preference for 3-year-old logs. *Treptoplatypus severini*, *X. crassiusculus*, and *X. germanus* preferred AL logs, while *H. seriatus* and *T. lineatum* preferred WB logs.

## 4. Discussion

Our results revealed that ambrosia beetles were mainly collected from logs in the first 2 years after felling (Table 1), thereby indicating that these beetles primarily utilized logs during the initial stages of wood decay. This is consistent with Kappes and Topp [42], who reported that scolytine beetles avoided logs that were felled three years prior. In total, six and nine species of ambrosia beetles were collected from AL and WB logs, respectively. Among the beetle species, *T. lineatum*, *E. validus*, *S. daimio*, and *S. mikado* exhibited a very small abundance. This may be due, in part, to their low population density. In spite of using ethanol-baited flight interception traps in the two plantations in each of the 3 years, in the present study, only 11 individuals of *S. mikado* were captured. None of the other three species were recorded (Peng Y et al., unpublished data), despite the success of trapping these four insect species with ethanol-baited traps in other locations in Japan [25,43,44,45]. Another possible cause is that AL and WB logs are not the major host materials of these insects. For instance, *T. lineatum* is usually considered a significant pest in various coniferous species and rarely colonizes hardwoods [46]. *Euwallacea validus* was highlighted as an indicator species of coniferous trees [45]. *Scolytoplatypus* species usually attack portions of trees with a fairly small diameter [47].

Although the two assessed host tree species were generally found to be utilized by a common group of ambrosia beetle species, the PERMANOVA results revealed that the species of tree had a significant influence on the species composition of ambrosia beetle communities colonizing AL and WB logs (Table 3), particularly with respect to the insect assemblages observed in 2016 or 2017 (Figure 5). In the first year, the ambrosia beetle assemblages of both tree species were dominated by *T. severini*, but the abundance of beetles were greater in AL than WB. We suspect that the difference in beetle abundance between AL and WB observed in 2016 could be attributable to the higher rate of *T. severini* and *X. germanus* colonization in AL (Figure 3B, D). However, during the second year of the study, beetles were observed to be more abundant in WB than in AL, although conversely the number of beetles captured using ethanol-baited traps was lower in the birch plantation than in the alder plantation (Peng Y et al., unpublished data). In 2017, *H. seriatus* became the most abundant species in WB logs, whereas beetle communities in the AL logs continued to be dominated by *T. severini*. The difference in beetle abundance between AL and WB observed in 2017 could be attributable to the greater rate of *H. seriatus* colonization in WB logs (Figure 3A). Unlike most other species in the Xyleborini tribe, *H. seriatus* usually bores its communal galleries in the inner bark of host trees and not the xylem [48,49]. During the dissection process in 2017, we observed that the inner bark of WB was still fresh and moister than that of AL. Therefore, it was reasonable that the majority of *H. seriatus* chose to colonize WB logs rather than AL logs. In 2018, only a small number of *T. severini* and *X. attenuatus* were collected from AL and WB logs, and we detected no significant difference in the structures of the beetle communities inhabiting AL and WB logs. We suspect that these observations reflect the fact that 3-year-old logs are probably too dry and/or over-colonized, thereby rendering them unsuitable hosts for ambrosia beetles; consequently, we recorded a marked reduction in attacks during 2018. The differences in the structures of the beetle communities inhabiting AL and WB logs may be related to their host species preferences in part, as our results indicated that *T. severini*, *X. crassiusculus*, and *X. germanus* preferred AL to WB, while *H. seriatus* and *T. lineatum* showed a preference for WB. However, the proximate causes for the host preferences of these insect species are not clear. Therefore, further study is required concerning the physical and chemical properties of the logs of the two tree species. In addition, the NMDS results revealed that the difference in the overall (3 years in total) beetle assemblages between the two tree species was smaller compared with the difference in either 2016 or 2017 (Figure 5). We conjecture that a possible reason for this result may be that AL logs deteriorate faster than WB logs. Firstly, after two years of deterioration, we observed that the bark of AL had become drier and more fragile than that of WB, and some parts of AL bark had been separated from the trunk or fallen off, whereas the bark of WB was wetter and remained intact (see Figure 1), which suggests that AL logs probably decay faster than WB logs, as tree species with rapidly decomposing bark often have trunks that decompose quickly [50]. The findings of a wood decomposition study conducted in North Sweden similarly indicated that the rate of alder (*A. incana*) decay was more pronounced than that of birch (*B. pubescens*) [51], although these tree species differed from those examined in the present study. Furthermore, we found that the timing of beetle species colonization tended to be earlier for AL than for WB, although individual beetle species appeared in the same sequence in the two tree species (Figure 4), which also supports the more rapid deterioration of AL timbers compared with that of WB. However, further studies on log decomposition are required to verify this hypothesis.

In warmer regions, some species of ambrosia beetles are known to spend more than one generation each year in colonized wood, or occasionally more than two generations in hot summers. For these species, it is probable that the number of attacks was underestimated in the present study, given that colonization by overwintering generations was not taken into consideration. However, *X. germanus* was the only species that showed twin peaks (in late May and mid-July, particularly in 2016), as indicated by the data obtained based on ethanol-baited trap captures in the two plantations (Peng Y et al., unpublished data). Although the sequence of appearance of individual ambrosia beetle species was the same for the two plant species, the timing of beetle colonization in AL logs was found to be slightly earlier than that in WB logs (Figure 4), thereby indicating that although the successional sequence of ambrosia beetles colonizing AL and WB logs was similar, the temporal patterns differed. On the basis of our observations indicating a similar sequence of ambrosia beetle species colonization of AL and WB logs, we were able to characterize the relative niches (early- to late-successional species) for each of the most abundant beetle species (e.g., *T. severini*, *Xylosandrus crassiusculus*, *Xylosandrus germanus*, *Xyleborinus attenuatus*, and *H. seriatus*) in the two plant species after felling. However, given the relatively low abundance of remaining species, it would be somewhat premature to speculate on their respective ecological niches based on the results of this study. During the relatively short succession period of ambrosia beetles on AL and WB logs, we suspect that *T. severini* and *X. crassiusculus* are early successional species that attack the freshest logs. Although *T. severini* was detected as an indicator species in the first two years of this study, it was conjectured that most of the *T. severini* galleries were probably colonized in 2016, given that the number of *T. severini* active galleries changed little from 2016 to 2017 but decreased significantly in 2018 (Figure 3D).

In the present study, the niche breadth was defined by a combination of attack timing and residence duration. Most ambrosia beetle species tend to remain in decaying timber for less than 1 year, as they typically complete their reproductive cycle (generally one generation) within a single year, and their offspring will fly away from the brooding material after completing development in the current year or during the following spring [5]. Very few species of ambrosia beetles are able to complete several generations in a given host, typically living trees [5]. Among the beetle species identified in our study, *T. severini* can sometimes remain in the same galleries for more than a single year if the quantity of wood and environmental conditions are suitable for the growth of their symbiotic fungus [52]. This would explain, at least in part, why *T. severini* showed the widest niche among colonizing beetles in our study, whereas other species showed narrower niches. Furthermore, *T. severini* is able to attack healthy living trees [52] and can only breed in undegraded host material with high moisture content, and not in over-decayed or dried wood [53]. Therefore, it was reasonable that *T. severini* was identified as a probable early-successional species that attacks AL and WB logs soon after cutting or even before cutting. Unlike most species in the tribe Xyleborini, which normally colonize unthrifty, cut, or broken trees, *X. crassiusculus* also attacks healthy living trees [54]. This species requires a high moisture content for successful reproduction [55]. Therefore, it is reasonable that *X. crassiusculus* mostly occurred in 2016, and thus was also considered as probable early-successional species. The other species identified in this study could not normally colonize healthy living trees but principally attacked dead or dying trees [46,47,49,56,57,58]. Among these species, *Xyleborinus attenuatus* and *H. seriatus* were identified as probable late-successional species, in that they showed a preference for 2-year-old logs of the two tree species (Table 4), whereas *Xylosandrus germanus*, which were mainly collected from 1-year-old AL logs and 2-year-old WB logs, is likely to be an intermediate species.

In line with expectation, we observed similarities in the ambrosia beetle faunas colonizing the two plant species. The data obtained using ethanol-baited traps indicated that ambrosia beetles in the two plantations mainly emerged from May to July, which suggests that host colonization typically occurred during this period (Peng Y et al., unpublished data). Therefore, it was thought that the samples collected in the fall would include most of the beetles that colonized the logs. Among the nine species of ambrosia beetles recorded in this study, six were found to colonize both AL and WB, including the four most abundant species. Previous studies have reported *H. seriatus* [41,59,60], *Xyleborinus attenuatus* [59,61,62], *Xylosandrus germanus* [59,63,64], *Xylosandrus crassiusculus* [54,59,63], *S. daimio* [59,60,65], *S. mikado* [60,65], and *T. lineatum* [66] in both alder (*Alnus*) and birch (*Betula*) trees. However, in our study, *S. mikado* and *T. lineatum* were found only in WB logs. This was probably due to their low population densities in our study sites and/or during our study period, and because AL and WB logs are not their common habitats, as discussed above. However, not all shared species in this study have been previously recorded in both alder and birch trees. For example, *T. severini* have been recorded in species belonging to the genus *Alnus* [60,67,68], but have not been recorded in the genus *Betula*. Consequently, to the best of our knowledge, this is the first study to report a species in the genus *Betula* being utilized as a host plant by *T. severini*.

## 5. Conclusions

Six and nine species of ambrosia beetles were collected from AL and WB logs, respectively, and they mainly appeared in the first two years after felling. In line with expectations, we recorded similarities in the ambrosia beetle assemblages colonizing AL and WB logs, with six beetle species, including the four most abundant species (*T. severini*, *H. seriatus*, *Xyleborinus attenuatus*, and *Xylosandrus germanus*), being detected in each of the two tree species. Moreover, we established that all beetle species colonizing AL and WB logs appeared in the same sequence, although the temporal patterns of colonization differed. Consequently, we detected differences in the species composition of beetle assemblages colonizing AL and WB logs in each of the first two years of the study, whereas over the entire study period, the difference in the overall beetle assemblages was smaller than that in either the first or second year after felling. We speculate that the different colonization patterns observed for the two tree species could be attributable to the rates at which the respective woods decay, although further studies are required to verify this assumption. The similar beetle faunas and sequences in which beetle species appeared indicate that the succession of beetles in the two plants was similar. Our results indicate that *T. severini* and *Xylosandrus crassiusculus* are probably early-successional species that colonize logs soon after felling, whereas *Xyleborinus attenuatus* and *H. seriatus* were identified as probable late-successional species that preferentially select older logs.

## Figures and Tables

**Table 1:** List of ambrosia beetle species found in alder and white birch logs in each sampling year. The number of active galleries is shown for each species. The values in brackets are the proportion of each species (%).

Subfamily	Tribe	Species	Abbreviation	Alder			White Birch		
				2016 (*n* = 129) ^1^	2017 (*n* = 75)	2018 (*n* = 17)	2016 (*n*= 129)	2017 (*n* = 77)	2018 (*n* = 18)
Platypodinae	Platypodini	*Treptoplatypus severini* (Blandford) ^2^	TS	3476 (77.99)	1342 (52.40)	19 (95.00)	953 (96.26)	880 (15.36)	43 (95.56)
Scolytinae	Xyleborini	*Xyleborinus attenautus* (Blandford)	XA	74 (1.66)	897 (35.03)	1 (5.00)	0	1514 (26.42)	2 (4.44)
		*Xylosandrus germanus* (Blandford)	XG	716 (16.06)	17 (0.66)	0	7 (0.71)	331 (5.78)	0
		*Heteroboripsseriatus* (Blandford) ^3^	HS	111 (2.49)	305 (11.91)	0	4 (0.40)	2984 (52.07)	0
		*Xylosandrus crassiusculus* (Motschulsky)	XC	74 (1.66)	0	0	4 (0.40)	1 (0.02)	0
		*Euwallacea validus* (Eichhoff)	EV	0	0	0	0	9 (0.16)	0
	Xyloterini	*Trypodendron lineatum* (Olivier)	TL	0	0	0	0	12 (0.21)	0
	Scolytoplatypodini	*Scolytoplatypus daimio* Blandford	SD	6 (0.13)	0	0	21 (2.12)	0	0
		*Scolytoplatypus mikado* Blandford	SM	0	0	0	1 (0.10)	0	0
**Total**				4457	2561	20	990	5731	45
**Number of species**				6	4	2	6	7	2

^1^ The number of billets of each tree species sampled in each year. ^2^ *Treptoplatypus severini* was transferred from *Platypus* by Beaver and Shih [40]. ^3^ *Heteroborips seriatus* was transferred from *Xyleborus* by Mandelshtam et al. [41].

**Table 2:** Effects of tree species and insect species on the niche center and breath of ambrosia beetles found in alder and white birch logs, analyzed using a generalized linear mixed model. For the tree species effect, alder was used in the base model with the insect species as the random effect. For the insect species effect, *Xyleborinus attenuatus* was used in the base model with the tree species as the random effect.

Factors	Predictors	Niche Center			Niche Breadth	
		Estimates	*p*-Value		Estimates	*p*-Value
Tree species	(Intercept)	1.39	<0.001		0.249	0.072
	White birch	0.321	0.063		−0.0726	0.620
Insect species	(Intercept)	1.963	<0.001		0.144	0.431
	*Euwallacea validus*	−0.073	0.818		−0.144	0.642
	*Treptoplatypus severini*	−0.563	0.067		0.557	0.066
	*Scolytoplatypus daimio*	−0.963	0.010		−0.144	0.571
	*Trypodendron lineatum*	−0.073	0.818		−0.144	0.642
	*Xyleborus seriatus*	−0.097	0.705		0.151	0.551
	*Xylosandrus crassiusculus*	−0.863	0.016		0.107	0.672
	*Xylosandrus germanus*	−0.462	0.115		−0.038	0.879

**Table 3:** Effects of tree species and sampling year on the community structure of ambrosia beetles found in alder and white birch logs from 2016 to 2018. The *R*-square and *p*-values were detected using PERMANOVAs.

Factors	*R* ^2^	*p*-Value
Tree species (SP)	0.0248	<0.001
Sampling year (YR)	0.3417	<0.001
SP * YR	0.1126	<0.001

**Table 4:** Correlation index of the relationship between the beetle species and tree species or sampling year, detected using indicator species analysis.

Insect Species	Tree Species		Alder			White Birch		
	Alder	White birch	2016	2017	2016 + 2017	2016	2017	2016 + 2017
*Treptoplatypus severini*	0.323 ***				0.465 ***			0.264 *
*Xyleborinus attenautus*				0.582 ***			0.536 ***	
*Xylosandrus germanus*	0.100 *		0.276 **				0.502 ***	
*Heteroborips seriatus*		0.235 ***		0.294 **			0.561 ***	
*Xylosandrus crassiusculus*	0.194 ***		0.319 **					
*Trypodendron lineatum*		0.128 **					0.254 **	

***: *p* < 0.001; **: *p* < 0.01; *: *p* < 0.05.

## Data Availability

The data presented in this study are available on request from the corresponding author. Insect specimens are stocked by N.K.

## References

[1] Vega F.E., Hofstetter R.W. (2015). Bark Beetles: Biology and Ecology of Native and Invasive Species.

[2] Kamata N., Esaki K., Kato K., Igeta Y., Wada K. (2002). Potential impact of global warming on deciduous oak dieback caused by ambrosia fungus *Raffaelea* sp. carried by ambrosia beetle *Platypus quercivorus* (Coleoptera: Platypodidae) in Japan. Bull. Entomol. Res..

[3] Fraedrich S.W., Harrington T.C., Rabaglia R.J., Ulyshen M.D., Mayfield A.E., Hanula J.L., Eickwort J.M., Miller D.R. (2008). A fungal symbiont of the redbay ambrosia beetle causes a lethal wilt in redbay and other Lauraceae in the southeastern United States. Plant Dis..

[4] Haack R.A., Rabaglia R.J., Peña J.E. (2013). Exotic bark and ambrosia beetles in the USA: Potential and current invaders. Potential Invasive Pests of Agricultural Crops.

[5] Wood S.P. (2007). Bark and Ambrosia Beetles of South America (Coleoptera: Scolytidae).

[6] Li Y., Simmons D.R., Bateman C.C., Short D.P.G., Kasson M.T., Rabaglia R.J., Hulcr J. (2015). New fungus-insect symbiosis: Culturing, molecular, and histological methods determine saprophytic polyporales mutualists of *Ambrosiodmus* ambrosia beetles. PLoS ONE.

[7] Byers J.A., Zhang Q., Liu T., Kang L. (2011). Chemical ecology of bark beetles in regard to search and selection of host trees. Recent Advances in Entomological Research: From Molecular Biology to Pest Management.

[8] Clements F.E. (1916). Plant Succession: An Analysis of the Development of Vegetation.

[9] McCullough H.A. (1948). Plant succession on fallen logs in a virgin spruce-fir forest. Ecology.

[10] Nakamura T., Suga H. (1995). Bryophyte succession on fallen logs in coniferous forests on Yaku-shima Island, southern Japan. Proc. Bryol. Soc. Jpn..

[11] Tang C.Q., Zhao M.H., Li X.S., Ohsawa M., Ou X.K. (2010). Secondary succession of plant communities in a subtropical mountainous region of SW China. Ecol. Res..

[12] Walker L.R., Moral R.D. (2003). Primary Succession and Ecosystem Rehabilitation.

[13] Austin M.P. (1977). Use of ordination and other multivariate descriptive methods to study succession. Vegetatio.

[14] Austin M.P. (1985). Continuum concept, ordination methods, and niche theory. Annu. Rev. Ecol. Syst..

[15] Kaufmann R. (2001). Invertebrate succession on an alpine glacier foreland. Ecology.

[16] Vater A.E. (2012). Insect and arachnid colonization on the Storbreen glacier foreland, Jotunheimen, Norway: Persistence of taxa sug-gests an alternative model of succession. Holocene.

[17] Vater A.E., Matthews J.A. (2013). Testing the ‘addition and persistence model’ of invertebrate succession in a subalpine glacier-foreland chronosequence: Fåbergstølsbreen, southern Norway. Holocene.

[18] Brändle M., Brandl R. (2006). Is the composition of phytophagous insects and parasitic fungi among trees predictable?. Oikos.

[19] Novotny V., Miller S.E., Baje L., Balagawi S., Basset Y., Cizek L., Craft K.J., Dem F., Drew R.A., Hulcr J. (2010). Guild-specific patterns of species richness and host specialization in plant-herbivore food webs from a tropical forest. J. Anim. Ecol..

[20] Watanabe K., Murakami M., Hirao T., Kamata N. (2014). Species diversity estimation of ambrosia and bark beetles in temperate mixed forests in Japan based on host phylogeny and specificity. Ecol. Res..

[21] Fukuda K., Kuraji K., Owari T., Yasumura N., Kamata N., Kamata N., Kuraji K., Owari T., Guan B.T. (2019). The University of Tokyo Forests. Developing a Network of Long-Term Research Field Stations to Monitor Environmental Changes and Ecosystem Responses in Asian Forests.

[22] Meteorology Division, Fundamental Data Development Committee, The University of Tokyo Forests (2018). Annual Report of Meteorological Observations in the University of Tokyo Forests, The University of Tokyo (January 2016—December 2016). Misc. Info. Univ. Tokyo For..

[23] Meteorology Division, Fundamental Data Development Committee, The University of Tokyo Forests (2019). Annual Report of Meteorological Observations in the University of Tokyo Forests, The University of Tokyo (January 2017—December 2017). Misc. Info. Univ. Tokyo For..

[24] Meteorology Division, Fundamental Data Development Committee, The University of Tokyo Forests (2020). Annual Report of Meteorological Observations in the University of Tokyo Forests, The University of Tokyo (January 2018—December 2018). Misc. Info. Univ. Tokyo For..

[25] Iidzuka H., Goto H., Yamasaki M., Osawa N. (2016). Wood-boring beetles (Coleoptera: Scolytidae, Platypodidae) captured in ethanol-baited traps in a natural forest in Japan. Appl. Entomol. Zool..

[26] Viloria Z., Villanueva R.T., Bessin R., O’Neal P., Ranger C.M., Dunwell W. (2021). Scolytinae in nursery and fruit crops of Western Kentucky and seasonal population patterns of four invasive ambrosia beetles. J. Entomol. Sci..

[27] Ruchin A.B., Egorov L.V., Khapugi A.A. (2021). Seasonal activity of Coleoptera attracted by fermental crown traps in forest ecosystems of Central Russia. Ecol. Quest..

[28] Netherer (2019). S.; Panassiti, B.; Pennerstorfer, J.; Matthews, B. Acute drought is an important driver of bark beetle infestation in Austrian Norway spruce stands. Front. For. Glob. Chang..

[29] Gandhi K.J., Cognato A.I., Lightle D.M., Mosley B.J., Nielsen D.G., Herms D.A. (2010). Species composition, seasonal activity, and semiochemical response of native and exotic bark and ambrosia beetles (Coleoptera: Curculionidae: Scolytinae) in northeastern Ohio. J. Econ. Entomol..

[30] Jackman S. (2020). Pscl: Classes and Methods for R Developed in the Political Science Computational Laboratory.

[31] Desmarais B.A., Harden J.J. (2013). Testing for zero inflation in count models: Bias correction for the Vuong test. Stata J..

[32] Hothorn T., Bretz F., Westfall P. (2008). Simultaneous inference in general parametric models. Biom. J..

[33] Yu S.X., Orlóci L. (1993). Species niche center: A useful ecological concept. Abstr. Bot..

[34] Colwell R.K., Futuyma D.J. (1971). On the measurement of niche breadth and overlap. Ecology.

[35] Zhang J.L. (2016). spaa: SPecies Association Analysis; R Package Version 0.2.2. https://CRAN.R-project.org/package=spaa.

[36] Pinheiro J., Bates D., DebRoy S., Sarkar D., R Core Team (2021). Nlme: Linear and Nonlinear Mixed Effects Models; R Package Version 3.1-152. https://CRAN.R-project.org/package=nlme.

[37] De Cáceres M., Legendre P. (2009). Associations between species and groups of sites: Indices and statistical inference. Ecology.

[38] Anderson M.J. (2017). Permutational multivariate analysis of variance (PERMANOVA). Wiley StatsRef Stat. Ref. Online.

[39] Oksanen J., Blanchet F.G., Friendly M., Kindt R., Legendre P., McGlinn D., Minchin P.R., O’Hara R.B., Simpson G.L., Solymos P. (2020). Vegan: Community Ecology Package; R Package Version 2.5-7. https://CRAN.R-project.org/package=vegan.

[40] Beaver R.A., Shih H.T. (2003). Checklist of Platypodidae (Coleoptera: Curculionoidea) from Taiwan. Plant Prot. Bull..

[41] Mandelshtam M.Y., Petrov A.V., Smith S.M., Cognato A.I. (2019). Resurrection of *Heteroborips* Reitter, 1913 (Coleoptera: Curculionidae: Scolytinae) from Synonymy with *Xyleborus* Eichhoff, 1864. Coleopts. Bull..

[42] Kappes H., Topp W. (2004). Emergence of Coleoptera from deadwood in a managed broadleaved forest in central Europe. Biodivers. Conserv..

[43] Ito M., Sato S., Kawasaki Y., Kajimura H. (2008). Ambrosia beetles captured with ethanol traps in Irazu-yama National Forest, Kochi Prefecture. Bull. FFPRI.

[44] Kamata N., Sanguansub S., Beaver R.A., Saito T., Hirao T. (2020). Investigating the factors influencing trap capture of bark and ambrosia beetles using long-term trapping data in a cool temperate forest in central Japan. J. Forest Res..

[45] Saito T., Goto H., Hirao T., Kamata N. (2013). Revision of a list of subfamily Scolytinae and Platypodinae captured by bait traps at the University of Tokyo Chichibu Forest in 1994–2003. Misc. Info. Univ. Tokyo For..

[46] Van Driesche R.G., LaForest J.H., Bargeron C.T., Reardon R.C., Herlihy M. (2013). Forest Pest Insects in North America: A Photographic Guide.

[47] Beaver R.A., Gebhardt H. (2006). A review of the Oriental species of *Scolytoplatypus* Schaufuss (Coleoptera, Curculionidae, Scolytinae). Dtsch. Entomol. Z..

[48] Nakashima T., Otomo T., Owada Y., Iizuka T. (1992). SEM observations on growing conditions of the fungi in the galleries of several ambrosia beetles: (Coleoptera Scolytidea and Platypodidae). J. Fac. Agric. Hokkaido Univ..

[49] Mandelshtam M.Y. (2006). New synonymies and new combinations in Scolytidae from the Kuril Archipelago and continental territories of the Russian Far East (Coleoptera). Zoosyst. Ross..

[50] Chang C., van Logtestijn R.S.P., Goudzwaard L., van Hal J., Zuo J., Hefting M., Sass-Klaassen U., Yang S., Sterck F.J., Poorter L. (2020). Methodology matters for comparing coarse wood and bark decay rates across tree species. Methods Ecol. Evol..

[51] Freschet G.T., Weedon J.T., Aerts R., van Hal J.R., Cornelissen J.H. (2012). Interspecific differences in wood decay rates: Insights from a new short-term method to study long-term wood decomposition. J. Ecol..

[52] Iidzuka H., Goto H., Yamasaki M., Osawa N. (2014). Ambrosia beetles (Curculionidae: Scolytinae and Platypodinae) on *Fagus crenata* Blume: Community structure, seasonal population trends and resource utilization patterns. Entomol. Sci..

[53] Atkinson T.H. (2000). Ambrosia beetles, *Platypus* spp. (Insecta: Coleoptera: Platypodidae). DPI Entomol. Circ. Univ. Florida.

[54] Kavčič A., de Groot M. (2017). Pest Risk Analysis for the Asian Ambrosia Beetle (Xylosandrus crassiusculus (Motschulsky, 1866)).

[55] Atkinson T.H., Foltz J.L., Wilkinson R.C., Mizell R.F. (2011). Granulate ambrosia beetle, *Xylosandrus crassiusculus* (Motschulsky) (Insecta: Coleoptera: Curculionidae: Scolytinae). DPI Entomol. Circ. Univ. Florida.

[56] EPPO (2020). EPPO Study on the Risk of Bark and Ambrosia Beetles Associated with Imported Non-Coniferous Wood. EPPO Paris. https://www.eppo.int/RESOURCES/eppo_publications.

[57] Björklund N., Boberg J. (2017). Rapid Pest Risk Analysis Xyleborinus attenuates.

[58] Galko J., Dzurenko M., Ranger C.M., Kulfan J., Kula E., Nikolov C., Zúbrik M., Zach P. (2019). Distribution, habitat preference, and management of the invasive ambrosia beetle *Xylosandrus germanus* (Coleoptera: Curculionidae, Scolytinae) in European forests with an emphasis on the West Carpathians. Forests.

[59] Kamata N., Iguchi K., Sanguansub S., Maneerat T. (2014). Infestation by insect borers on *Betula maximowicziana* following successive years of severe defoliation by *Caligula japonica* (Lepidoptera: Saturniidae) with special reference to *Xyleborus seriatus* Blandford (Coleoptera: Curculionidae: Scolytinae). Forest Pest..

[60] Wood S.L., Bright D. (1992). A catalog of Scolytidae and Platypodidae (Coleoptera), Part 2. Great Basin Nat. Mem..

[61] Nikulina T., Mandelshtam M., Petrov A., Nazarenko V., Yunakov N. (2015). A survey of the weevils of Ukraine. Bark and ambrosia beetles (Coleoptera: Curculionidae: Platypodinae and Scolytinae). Zootaxa.

[62] Borowski J., Piętka J., Szczepkowski A. (2012). Insects found on black alder *Alnus glutinosa* (L.) Gaertn. when stands are dying back. Forest Res. Pap..

[63] Dole S.A., Cognato A.I. (2010). Phylogenetic revision of *Xylosandrus* Reitter (Coleoptera: Curculionidae: Scolytinae: Xyleborina). Proc. Calif. Acad. Sci..

[64] Weber B.C., McPherson J.E. (1983). World list of host plants of *Xylosandrus germanus* (Blandford) (Coleoptera: Scolytidae). Coleopt. Bull..

[65] Nobuchi A. (1980). The ambrosia beetles of the subfamily Scolytoplatypinae (Coleoptera, Scolytidae) in Japan. Kontyû Tokyo.

[66] Borden J.H., Berryman A.A. (1988). The striped ambrosia beetle. Dynamics of Forest Insect Populations: Patterns, Causes, Implications.

[67] Murayama J. (1925). On the Platypodidae of Formosa. J. Coll. Agric. Hokkaido Imperial Univ..

[68] Nobuchi A. (1973). The Platypodidae of Japan (Coleoptera). Bull. Gov. For. Exp. Sta..

